# Dual infections of two carbapenemase-producing *Acinetobacter baumannii* clinical strains isolated from the same blood culture sample of a patient in Iran

**DOI:** 10.1186/s13756-018-0329-x

**Published:** 2018-03-10

**Authors:** Linda Hadjadj, Saeed Shoja, Seydina M. Diene, Jean-Marc Rolain

**Affiliations:** 1 Aix Marseille Univ, IRD, APHM, MEPHI, IHU-Méditerranée Infection, Marseille, France; 2 Infectious and Tropical Diseases Research Center, Hormozgan Health Institute, Hormozgan University of Medical Sciences, Bandar Abbas, IR Iran

**Keywords:** *Acinetobacter baumannii*, Carbapenemase, Genome, Iran

## Abstract

In this study, the draft genome sequences of two different carbapenem-resistant *Acinetobacter baumannii* clinical strains isolated from the same blood culture sample of an Iranian patient were determined. The strain *A. baumannii* 554S harbouring *bla*_oxa72_ gene belonged to ST 307 whereas *A. baumannii* 554L carrying *bla*_oxa23_ gene belonged to ST 2. We found that this sample contains two different isolates of *A. baumannii,* each phenotypically and genetically different.

## Introduction

*Acinetobacter baumannii* is one of the most common opportunistic Gram-negative bacterial pathogen causing nosocomial infections. The worldwide emergence of multidrug-resistant *A. baumannii* (MDR-AB), especially carbapenem-resistant isolates, has been reported [1]. In Iran, the prevalence of MDR-AB is currently high and on the rise, resulting in increasing mortality and morbidity rates among patients [2].

## Case report

**Fig. 1:**
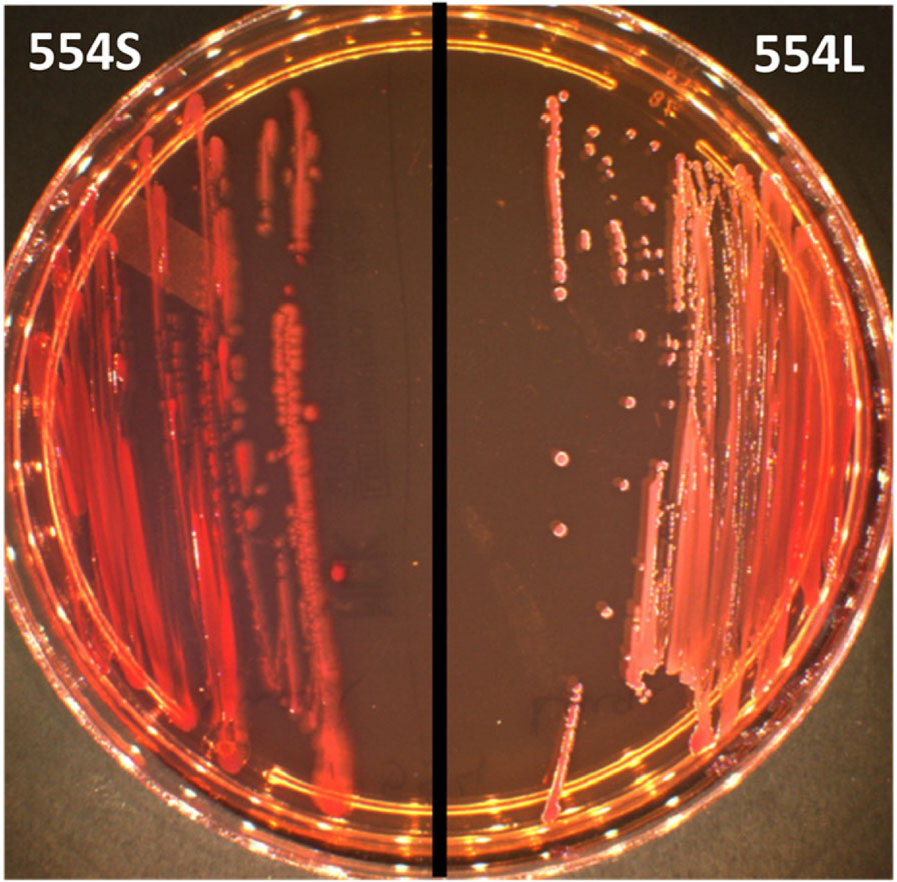
Isolation of *A. baumannii* strains 554S and 554L on Mac Conkey plate: strain 554S on left and strain 554L on right

In this study, *A. baumannii* strains 554S and 554L were isolated from the blood sample of a 10-year-old girl with a burn injury who was hospitalized at Taleghani Burn Hospital in Ahvaz, southwest Iran, on July 25, 2012. On July 27, she became febrile, so a blood culture was realized. Because of the delay between admission and positive culture, we considered that the infection was hospital-acquired. From July 27 to July 31, she was treated empirically with 20 mg of amikacin and 1 g of ceftazidime. Then, on August 1, her antibiotic treatment was replaced with 500 mg of meropenem and 2 g/250 mg of piperacillin-tazobactam. From August 3 to August 11, this treatment was replaced with 500,000 IU of colistin. Then, the patient moved hospitals and sadly died on August 21, 2012. Thus, we believe that the patient eventually died from the burn injury and not from the infection with these bacteria. After the blood sample was plated on chocolate agar, two different colonies with different morphological aspects were observed: one small and one large colony (Fig. 1). The two isolates were identified by MALDI-TOF (matrix-assisted laser desorption/ionization time-of-flight mass spectrometry; Microflex, Bruker Daltonics, Bremen, Germany) as *Acinetobacter baumannii* strains. Antibiotic susceptibility testing (AST) was performed by the disc diffusion method and interpreted according to the European Committee on Antimicrobial Susceptibility Testing guidelines (http://www.eucast.org/). The two isolates were resistant to carbapenems but full AST revealed that the strains do not have the same profile. The isolate 554L harboring *bla*_OXA-23_ was resistant to all antimicrobials tested with the exception of tigecycline and colistin, whereas the isolate 554S harboring *bla*_OXA-72_ was also susceptible to doxycycline and tetracycline (Table 1).

**Table 1:** Characteristics of *A. baumannii* strains 554S and 554L analyzed in this study

Strains	ST	Size (bp)	GC%	Genomic features	Susceptible AST phenotype	Resistant AST phenotype	Resistant genes	Genbank accession number
554S	307	4,056,106	39	3884 CDS,8 rRNAs, 64tRNAs	TIG, CT, DOX, TET	AMX, AMC, TCC, FOX, CTX, CRO, IMP, ETP, CIP, AZT, AK, TOB, GEN, RA, SXT	*bla*_MBL_, *bla*_A1_, *bla*_A2,_ *bla*_ADC-76_, ***bla***_**OXA-72**_, *bla*_OXA-235_, *bla*_OXA-64_, *arm*A, *aphA6, mphE, msrE, sul2*	NKXP00000000
554L	2	3,922,359	39.1	3689 CDS,8 rRNAs, 62 tRNAs	TIG, CT	AMX, AMC, TCC, FOX, CTX, CRO, IMP, ETP, CIP, AZT, AK, TOB, GEN, RA, SXT, DOX, TET	*bla*_MBL_, *bla*_A1_, *bla*_A2,_ *bla*_ADC-73_, *bla*_TEM-1D_, ***bla***_**OXA-23**_, *bla*_OXA-66_, *arm*A, *aph3”, Ia, aphA6, mphE, msrE, sul2, str A, strB, tetB*	NKXQ00000000

CDS: Coding DNA Sequence, AMX: Amoxicillin, AMC: Amoxicillin/clavulanic acid, TCC: Ticarcillin/clavulanic acid, FOX: Cefoxitin, CTX: Cefotaxime, CRO: Ceftriaxone, IMP: Imipenem, ETP: Ertapenem, CIP: Ciprofloxacin, AZT: Aztreonam, AK: Amikacin, TOB: Tobramycin, GEN: Gentamycin, RA: Rifampicin, SXT: Sulfamethoxazole/trimethoprim, DOX: Doxycycline, TET: Tetracyclin, TIG: Tigecycline, CT: Colistin

Genomic DNA (gDNA) of *A. baumannii* strains 554S and 554L were sequenced on a MiSeq sequencer (Illumina Inc., San Diego, CA, USA) using the paired-end strategy. Raw reads were assembled with A5-miseq software [3]. Genomes annotation was performed by Rapid Annotation using Subsystem Technology (RAST) [4]. tRNA gene detection was performed using the tRNAScanSE 2.0 tool [5], whereas ribosomal RNA genes were detected using RNAmmer [6]. The sequence type (ST) of strains was analyzed by the MLST 1.8 server of the Center for Genetic Epidemiology. Antimicrobial resistance gene profiling was performed in silico using ARG-ANNOT [7]. Further bioinformatic analysis, such as identification of genomic islands, prophage sequences, clustered regularly interspaced short palindromic repeat (CRISPR) sequences, secondary metabolite gene clusters and plasmid presence were predicted by employing IslandViewer, PHASTER, CRISPRFinder, antiSMASH and Plasmid finder tools, respectively. The percentage of similarity between strains was calculated by pairwise comparison of their Average Nucleotide Identity based on Blast (ANIb) by Jspecies software [8].

The draft genome of 554S had 386 scaffolds that comprised 4,056,106 bp with a 39% G + C content. Similarly, the 554L draft genome was composed of 580 scaffolds, having a total size of 3,922,359 bp long with 39.1% G + C content. The draft genomes of *A. baumannii* strains 554S and 554L contained 3884/3689 coding sequences, 8/8 rRNAs, 64/62 tRNAs, respectively. In silico MLST analysis using the Pasteur Institute typing scheme revealed that strains 554S and 554L belong to ST 307 and ST 2, respectively. The strain 554S had 97.1% of similarity with the strain 554L. ARG-ANNOT result analysis revealed that the genomes of strains 554S and 554L encoded 12 and 16 antimicrobial resistance genes, respectively. Genes that harbored resistance to beta-lactams (*bla*_MBL_, *bla*_A1_, *bla*_A2_), aminoglycosides (*armA*, *aphA6*), macrolides (*mphE*, *msrE*) and sulfamides (*sul2*) were detected in both genomes. Moreover, in the 554S genome we found genes *bla*_ADC-76_, *bla*_OXA-72_, *bla*_OXA-235_, *bla*_OXA-64,_ while the genes *bla*_ADC-73_, *bla*_TEM-1D_, *bla*_OXA-23_, *bla*_OXA-66_, *aph3”Ia*, *strA*, *strB* and *tetB* were unique to the 554L genome (Table 1). Both strains presented the disrupted ATPase encoding comM gene. The genome of the isolate 554S contained at least 28 genomics islands and one complete prophage sequence. Also, three CRISPR sequences could be predicted. For the genome of the isolate 554L, 23 genomic islands and one incomplete prophage sequence were found but no CRISPR sequences. The presence of three putative secondary metabolite gene clusters, including arylpolyene, siderophore and non-ribosomal peptide synthetase (NRPS) gene clusters, could also be predicted in both genomes whereas hserlactone-arylpolyene was predicted only in the 554L genome. Neither strain carried a plasmid.

## Discussion

In summary, we reported the draft genome sequences of two clinical MDR-AB strains in Iran. *A. baumannii* is known with its high resistance to carbapenems worldwide [1, 9]. In a national Iranian study, the mean prevalence of MDR-AB was 71% during the period 2010–2015 [2]. In the Taleghani Burn Hospital of our study, this prevalence increased with 92.5% of *A. baumannii* carbapenem resistant [10]. Carbapenem resistance in *A. baumannii* was principally due to the production of oxacillinases including OXA-23, OXA-24, OXA-58 enzymes, and the New Delhi Metallo-β-lactamase 1 (NDM-1) [1]. OXA-23 enzymes were predominant among MDR-AB in several regions worldwide [9]. The international clone II corresponding to ST 2 of the Pasteur MLST scheme was associated with nosocomial outbreaks and had spread globally [9, 11]. As with Europe, the spread of MDR-AB clonage lineage II producing *bla*_oxa23_-like and *bla*_oxa24_-like was described in Iran [12]. Our results confirmed these studies with the description of one MDR-AB strain 554L carrying *bla*_oxa23_ gene belonging to ST 2 and the second strain 554S harbouring *bla*_oxa72_ (*bla*_oxa24_-like) gene belonging to ST 307. Although isolated from the same blood culture sample, these two strains were phenotypically and genetically different. The different ST and the weak percentage of similarity between these strains confirmed that we had two different clones of *A. baumannii* in the same sample. These isolates were resistant to several antibiotics and only tigecycline and colistin were effective on both strains. Each strain carried different resistance genes, with the *bla*_OXA-72_ encoding carbapenem resistance gene found in the isolate 554S and the *bla*_OXA-23_ gene present in the strain 554L. The co-occurrence of different carbapenemase genes in the same *Acinetobacter* strain has already been described [13]. However, few genome sequence analyses explain this phenomenon. We could observe one carbapenemase encoding gene in a chromosome and one in a plasmid [14], or both in a chromosome [13]. In our case, without a careful observation of the colonies’ morphological aspect, we might conclude the presence of one strain of *A. baumannii* harboring two carbapenemases genes (*bla*_OXA-23_ and *bla*_OXA-72_), whereas we had two different *A. baumannii* strains, each harboring one carbapenemase gene. Hence, this study has revealed the possibility that double carbapenemase producers previously reported in the literature could in fact be a dual infection. Further work is warranted to understand if this phenomenon is common in human infections. These data also revealed molecular mechanisms contributing to bacterial drug resistance dissemination and expanded our understanding of the genomic features of MDR-AB in Iran. To our knowledge, this was the first draft genome sequences of MDR-AB isolates in Iran.

These Whole Genome Shotgun projects have been deposited in DDBJ/EMBL/GenBank under the sequence accession numbers NKXP00000000 and NKXQ00000000 for strains 554S and 554L, respectively.

## Abbreviations

ANIb: Average Nucleotide Identity based on Blast
AST: antibiotic susceptibility testing
CRISPR: clustered regularly interspaced short palindromic repeat
gDNA: genomic DNA
MALDI-TOF: matrix-assisted laser desorption and ionization time-of-flight mass spectrometry
MDR-AB: multidrug-resistant *Acinetobacter baumannii*
MLST: multilocus sequence typing
RAST: Rapid Annotation using Subsystem Technology
ST: sequence type
